# Breast cancer trends in women in Cyprus: a population-based study between 2004-2017

**DOI:** 10.1093/eurpub/ckac131.141

**Published:** 2022-10-25

**Authors:** A Quattrocchi, CA Demetriou, OA Cory, B Saad, C Constantinou, Y Marcou, A Demetriou, V Scoutellas, O Kolokotroni

**Affiliations:** 1 Primary Care and Population Health, University of Nicosia Medical School, Nicosia, Cyprus; 2 University of Nicosia Medical School, Nicosia, Cyprus; 3 Basic and Clinical Sciences, University of Nicosia Medical School, Nicosia, Cyprus; 4 Bank of Cyprus Oncology Center, Nicosia, Cyprus; 5 Health Monitoring Unit, Ministry of Health, Nicosia, Cyprus; 6 School of Health Sciences, Cyprus University of Technology, Limassol, Cyprus

## Abstract

**Background:**

In Cyprus, breast cancer (BC) is the first in incidence and second in mortality cancer in women. A national screening programme (NSP), targeting women 50-69 years, was introduced in 2007. The aim of this study is to provide a better understanding of cancer trends.

**Methods:**

Data from the national population-based Cyprus Cancer Registry on adult women diagnosed with BC between 2004-2017 with follow-up until 2019 were analysed as follows: Joinpoint regression for age-adjusted (overall and by tumor stage at diagnosis - TSD) and age-specific rates (<50, 50-59, 60-69, 70-79, ≥ 80) incidence and mortality rates; 5-year age-adjusted Net Survival (NS) rates, overall and by TSD. TSD was categorised as localised, regional, and distant.

**Results:**

Age-adjusted incidence rate increased from 135.3 (2004) to 153.2 (2017) per 100,000, with an annual percentage change (APC) of 1.1% (95%CI: 0.4-1.9). The greatest increase was in the age groups ≥70 years. A positive time trend was found for localized cancers between 2006-2017, while for all other stages nonsignificant trends were detected. Age-adjusted mortality rate increased from 37.0 (2004) to 50.0 (2019) per 100,000 (APC: 2.7%; 95%CI: 1.9-9.4). Significant increases in mortality rates were detected in the age groups ≥70 years. By TSD, increased rates were found at localised and regional stages, however smaller increases were detected since 2007. NS rates for the most recent period (2014-2017) was 93% for localized, 81% for regional, and 32% for distant and did not significantly improve compared to the previous years.

**Conclusions:**

Trends in BC incidence continues to increase, especially in the older age groups and for early-stage cancers. As expected, since the introduction of the NSP, the incidence of localised cancers increased whilst the incidence of advanced stage cancer decreased, albeit non-significantly. Survival trends did not change but mortality rates for localised and regional cancers increased at a slower pace.

**Key messages:**

• The introduction of the national screening programme may have played an important role in the increasing BC incidence trends.

• Despite survival rates not improving since the introduction of the national screening programme, mortality rates for early-stage cancers show a less steep increase.

